# An Efficient and Intelligent Detection Method for Fabric Defects based on Improved YOLOv5

**DOI:** 10.3390/s23010097

**Published:** 2022-12-22

**Authors:** Guijuan Lin, Keyu Liu, Xuke Xia, Ruopeng Yan

**Affiliations:** 1 School of Mechanical and Automotive Engineering, Xiamen University of Technology, Xiamen 361024, China; 2 Quanzhou Institute of Equipment Manufacturing, Haixi Institute, Chinese Academy of Sciences, Jinjiang 362216, China

**Keywords:** deep learning, computer vision, fabric detection, Swin Transformer, YOLOv5

## Abstract

Limited by computing resources of embedded devices, there are problems in the field of fabric defect detection, including small defect size, extremely unbalanced aspect ratio of defect size, and slow detection speed. To address these problems, a sliding window multihead self-attention mechanism is proposed for the detection of small targets, and the Swin Transformer module is introduced to replace the main module in the original YOLOv5 algorithm. First, to reduce the distance between several scales, the weighted bidirectional feature network is employed on embedded devices. In addition, it is helpful to improve the perception of small-target faults by incorporating a detection layer to achieve four-scale detection. At last, to improve the learning of positive sample instances and lower the missed detection rate, the generalized focal loss function is finally implemented on YOLOv5. Experimental results show that the accuracy of the improved algorithm on the fabric dataset reaches 85.6%, and the mAP is increased by 4.2% to 76.5%, which meets the requirements for real-time detection on embedded devices.

## 1. Introduction

Fabric defect detection is a core and important link in the entire textile quality production process. At present, the detection effect of existing methods for some small and widely distributed defects cannot meet the requirements of manufacturers [1]. Thus, it is of great importance to use accurate and efficient detection methods to improve the detection and identification of fabric defects.

In the field of fabric defect detection, traditional image processing methods are only suitable for detecting solid-color fabrics [2,3,4,5,6]. For fabrics with complex texture patterns, such as printed and jacquard fabrics, defect types are difficult to distinguish, especially for the detection of small defect targets. Traditional visual processing methods have been difficult to meet the needs of enterprises. Combining traditional vision techniques with deep learning for appearance defect detection of various objects has achieved considerable results. Reference [7] applies supervised learning to fabric defect detection by extracting effective features. Reference [8] trains a stacked denoising autoencoder based on Fisher’s criterion by using defective and nondefective samples, and uses the residual threshold to locate defects. With the development of the multiscale detection network, the detection methods of fabric multidefects have been proposed one after another. Zhang et al. [9] proposed a method for automatic location of fabric defects based on YOLO, which can meet the classification and detection of colored fabrics. Wang [10] proposed a detection algorithm based on the DeeplabV3+ model, which used the advantages of multiscale target detection and improved detection accuracy while reducing the network model parameters, as well as the ability to detect small-sized targets. Good results are obtained in the defect dataset.

In practical production applications, there is the problem of data imbalance in the defects of fabrics. For example, the number of fabric defect samples is small, some span of fabric defect sizes is large, and the number of small defects is large. Reference [11] divides and synthesizes multiple reconstruction residual images to obtain new defect detection results. This method can reduce the difficulty of collecting defect samples. Huang et al. [12] first take untrained fabric data as input. The output of the segmentation model is then used as raw material for the decision model. This approach requires only a few defect samples and can train a more accurate detection model.

In this study, we propose an efficient and intelligent detection method for fabric defects. The main contributions of this paper are as follows:

1. Based on the Transformer structure, we optimize the YOLOv5 v6.1 algorithm with the Swin Transformer as the backbone, and the introduction of a multiwindow sliding self-attention mechanism complements the convolutional network to improve classification accuracy.
2. In the neck layer, the BiFPN is used to replace the original FPN to enhance the fusion of semantic information between different layers, and a small-target detection layer is added to improve the detection effect of the model on small targets.
3. We introduce the generalized focal loss function to enhance the model’s instance learning of positive samples, in order to alleviate the problems caused by the imbalance of fabric samples.
4. Finally, we conducted ablation experiments and an in-depth analysis of the impact of the above-mentioned improved methods and several attention mechanisms on detection accuracy and real-time performance. Our proposed method outperforms current popular object detection models on a self-created fabric dataset.

In the remainder of this paper, the second section summarizes the YOLOv5 algorithm and the optimization method of this paper. Section 3 presents the fabric dataset, experimental details, and concrete results. The last section is the conclusion of this paper and the prospect of follow-up work.

## 2. Materials and Methods

There are generally two target detection methods at present: two stage and one stage [13]. Two stage first generates a series of sample candidate boxes through the algorithm, and then performs classification through a series of convolution operations. Mainstream two-stage algorithms include the R-CNN network proposed by Girshick [14] in 2014. The two-stage network is characterized by high accuracy of positioning and detection, but due to the complex network structure and poor real-time performance, it is not effective for rapid detection in the industry. One-stage methods do not need to select the sample candidate frame. They can directly obtain the coordinates and type of the target, which not only has better real-time performance, but also has advantages in small-target detection [15,16,17,18].

### 2.1. Structure of the YOLOv5

You only look once [19] (YOLO) is a single-stage target detection algorithm based on full convolution. YOLO [20] can predict the entire picture and give all the detection results at one time. The YOLOv5 algorithm is the fifth version of the YOLO algorithm launched by the Ultralytics LLC team. Its model has the characteristics of simplicity, speed, and portability.

**Figure 1:**
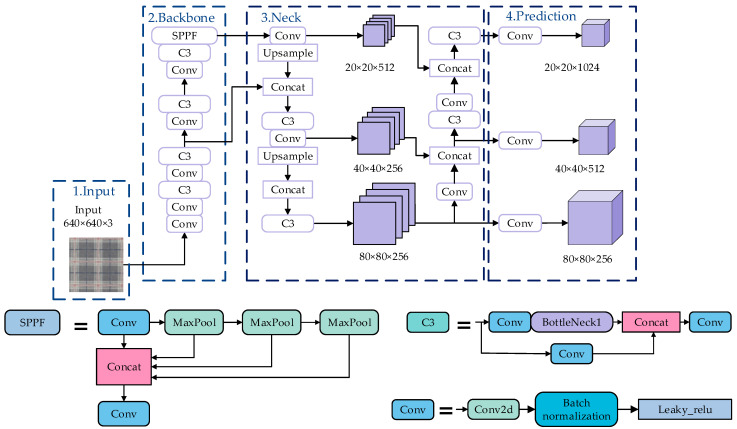
Network structure for YOLOv5.

The YOLOv5 algorithm framework consists of the input, the backbone, the neck, and the prediction. The structure diagram is shown in Figure 1.

The input of YOLOv5 mainly uses two sections: mosaic data enhancement and adaptive anchor frame. Mosaic data enhancement can read four pictures at a time; randomly scale, crop, and arrange each picture; and finally, randomly splice them together. This can greatly enrich the number of datasets and make the entire network more robust. For pictures of different sizes, first, the size of the input image is adjusted to a uniform set size, and in the process of scaling and filling, the original samples are adaptively populated and then sent to the backbone network.

The backbone network consists of a Conv structure and C3 structure. The CSP [21] structure solves the problem of excessive calculation in reasoning from the perspective of model structure design. The role of the SPPF layer increases the receptive field through convolution operations and maximum pooling. This module can strengthen the nonlinear expression capability.

The feature fusion layer neck adopts the structure of PAN + FPN [22], which fuses different detection layers with the main feature layer. The FPN only enhances the transmission of semantic information, but the ability to transmit low-level positioning information is not strong. On the basis of the FPN, PAN adds a bottom-up pyramid through a 3 × 3 convolution to enhance the transmission of positioning information. On the prediction side, this module uses GIoU [23] to calculate the loss value of the bounding box [24].

### 2.2. Swin Transformer Model

**Figure 2:**
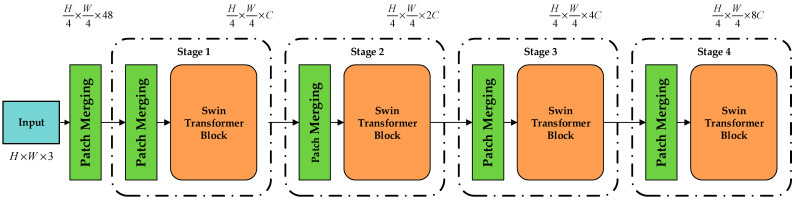
Swin Transformer network structure.

The Swin Transformer [25] model consists of multilayer perceptron (MLP), layer normalization (LM), window multihead self-attention (W-MSA), and sliding window multihead self-attention(SW-MSA). The structure of the Swin Transformer is similar to the traditional residual structure, so it can be directly used in convolutional networks. The Swin Transformer’s structure in the backbone layer is shown in Figure 2.

The workflow is as follows: (1) The Swin Transformer first inputs an RGB three-channel image of size H × W. It divides the input image into 4 × 4 patches through the segmentation layer, and its feature dimension is divided into h/4 × h/4. (2) In the process of Stage 1, the dimension of the output is changed to C through linear embedding, and then dispatches the Transformer block for Stage 2. (3) The purpose of Stage 2 to Stage 4 is the same. After the 2 × 2 adjacent blocks are spliced through image block patch merging, the spliced high-dimensional features are then reduced in dimension through a convolution, and the output dimension becomes 2C. Then, Stage 3 is repeated 6 times, and the output after dimensionality reduction is 4C. Stage 4 is repeated twice, and the output dimension becomes 8C.

**Figure 3:**
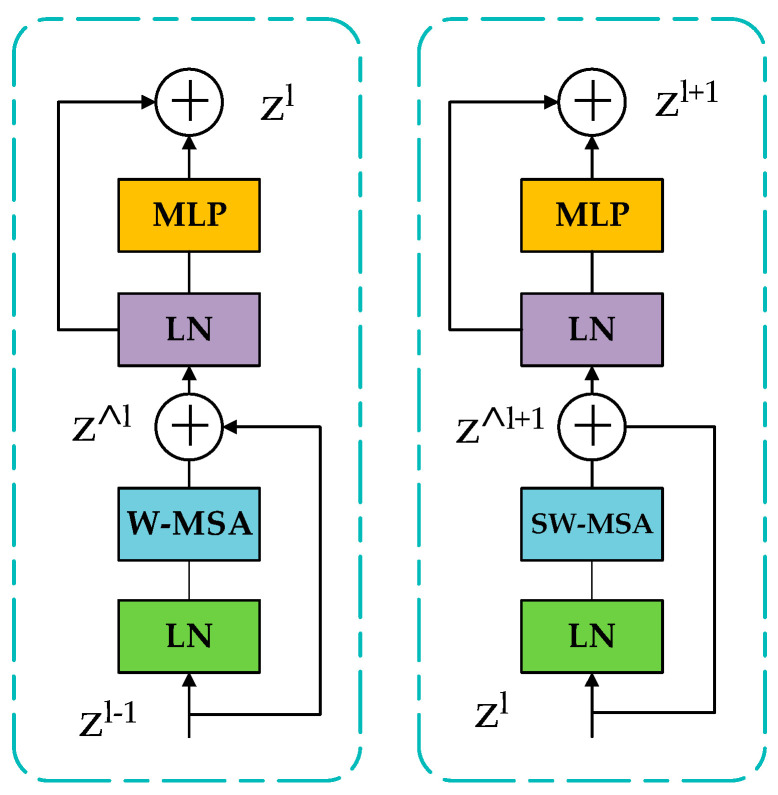
Swin Transformer’s backbone network.

The standard Transformer encoder consists of a multihead self-attention mechanism and a multilayer perceptron. It uses the layer norm at the beginning of the module, and then uses residual connections between each module. Figure 3 shows its structure. For the Swin Transformer backbone, the layer and calculation formula is:

ẑ^l^ = W − MSA (LN (z^l − 1^)) + z^l − 1^ (1)

z^l^ = MLP(LN(ẑ^l^)) + ẑ^l^ (2)

ẑ^l + 1^ = SW − MSA (LN (z^l^)) + z^l^ (3)

z^l + 1^ = MLP(LN(ẑ^l + 1^)) + ẑ^l + 1^ (4)

In Formulas (1) to (4) above, ẑ represents the feature output by MLP, and z^l^ is the output feature of W-MAS. W-SMA is a traditional window-partitioned self-attention mechanism, while SW-MSA represents a multihead self-attention mechanism using shifted window partitions. By introducing adjacent nonoverlapping windows in the upper layer, the connection between each layer is increased and the classification accuracy is improved.

### 2.3. Multiwindow Sliding Self-Attention Mechanism

The self-attention in Transformer is the key module of the algorithm. The traditional Transformer structure uses a global self-attention mechanism [26], which greatly increases the amount of computation. The self-attention mechanism based on the local window proposed by the Swin Transformer can make the computing window evenly divide the image in a nonoverlapping manner, and the computational complexity of the window based on H × W image blocks is much smaller than that of the global attention mechanism.

**Figure 4:**
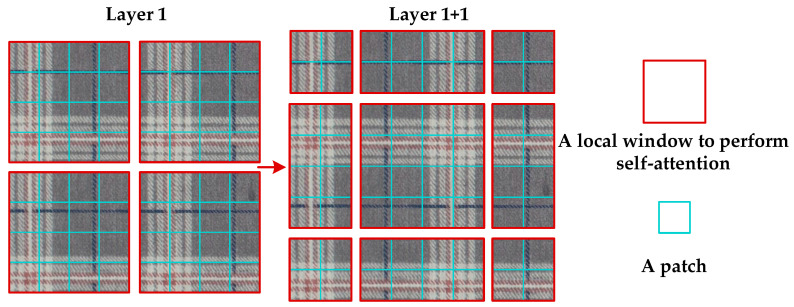
SW-MSA window moves.

SW-MSA is not limited to different windows for information exchange. As shown in Figure 4, in the first layer, the normal window division method, but at the l + 1 layer, the door division is moved, and the use of the mobile mouthpiece splitting method makes it possible to connect with each other without overlapping the entrance, greatly increasing the reception area of the mouth and increasing the special delivery ability.

### 2.4. Multiscale Feature Fusion Feature Pyramid Network

In a convolutional neural network, the feature maps obtained by convolutional layers with different parameters contain the feature information of different targets. The feature map obtained after deep convolution has higher resolution, mainly contains position information, but lacks semantic information, while the content obtained by shallow convolution is just the opposite of the former. Therefore, it is necessary to fuse the feature information of the deep feature map and the shallow feature map. The original YOLOv5 algorithm bidirectionally fuses the FPN and PAN in the neck layer to extract the information from different feature layers.

**Figure 5:**
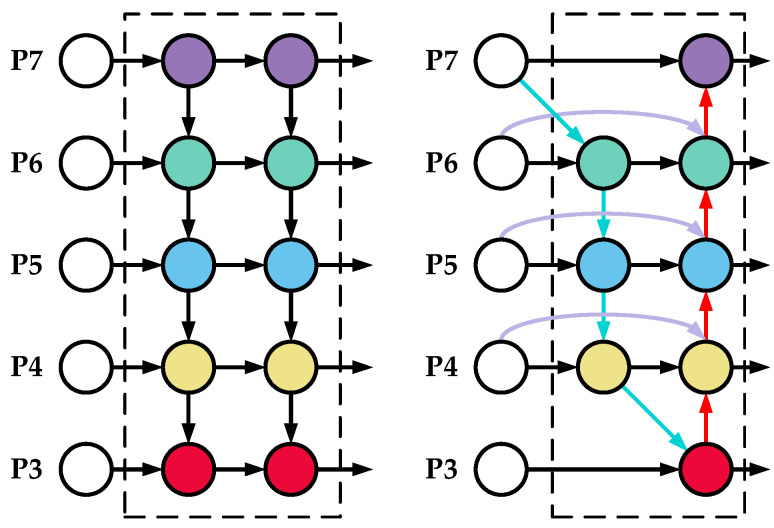
Structure of BiFPN.

The size of some defects in the fabric is too small, which will cause the feature information extracted by YOLOv5 to ignore the small defect information. In order to strengthen the feature fusion between different scales and increase the detection accuracy, this paper introduces a weighted bidirectional feature network [27] (bidirectional feature network), which uses weighted feature fusion and cross-scale connections to obtain multiple levels. The global features of semantic information can strengthen the recognition accuracy of small object defects. The structure of the BiFPN is shown in Figure 5.

The model is used for multidefect detection, and the target defect size on the fabric is not the same. Compared with common target detection algorithms (feature pyramid networks (FPNs)), the BiFPN uses skip connections to lighten the network. An attention mechanism is added to extract deeper feature information. Feature fusion is performed bidirectionally through upsampling and downsampling to improve the feature fusion effect between different layers.

Feature maps of 3 different sizes are used in the original YOLOv5 network results to detect objects with inconsistent sizes. When we set the input image size to 640 × 640, after a series of convolution and sampling operations, the size of the output detection feature map is 20 × 20, 40 × 40, and 80 × 80, which can detect 32 × 32, 16 × 16, and 8 × 8 targets respectively.

**Figure 6:**
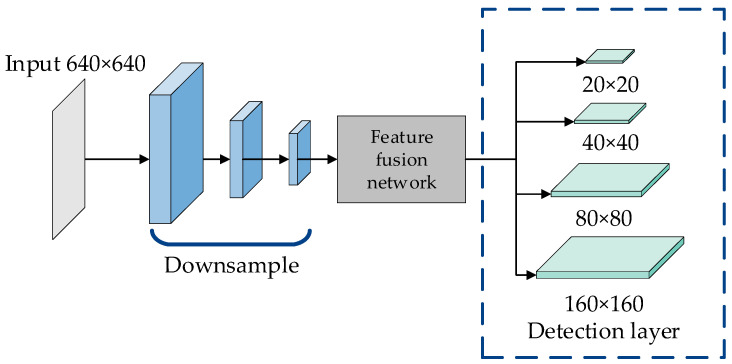
Improved four-scale detection layer.

Considering that the pixel size occupied by the small fabric defect targets is extremely small, to further strengthen the detection accuracy of small objects, we improve the algorithm to increase the number of upsamples to improve the lower-level feature information. Four-scale detection is formed by adding a 160 × 160 detection layer. The improved detection was shown in Figure 6. For the 640 × 640 input image, the detection layer can detect the 4 × 4 size target, which further improves the ability to extract small pixel defects in the fabric image.

### 2.5. Improvement of Loss Function

The loss function is obtained by calculating the error between the real frame and the predicted frame of the positive sample. The loss function of YOLOv5 mainly includes the classification loss function, the localization loss function, and the confidence loss function. In fabric defect detection, the area where the defect is located accounts for a small proportion of the entire fabric image. During training, we regard the area containing the defect as a positive sample and the normal area as a negative sample, which will lead to defective samples. The number of defects is much lower than the normal number of samples in the image area of the fabric. The loss value obtained is mostly the background loss of negative samples. Therefore, focal loss [28] is often cited to balance the number of foreground and background detection samples.

However, there are two problems with focal loss. One is that the focal loss function calculates the positioning quality score (IoU score) and the classification score (classification score) separately during training, but the two are comprehensively multiplied during testing as nonmaximum suppression (nonmaximum suppression (NMS)) sorting basis. This method will lead to a large error between training and testing, which will lead to a decrease in the performance of the detection model and ultimately affect the detection accuracy.

To solve the above problems, the IoU score is merged with the classification score. Since the combined category label becomes a continuous value of 0–1, and focal loss only calculates discrete labels of 0 or 1, this paper introduces generalized focal loss [29] to realize the fused representation of the IoU score and classification score. Its calculation formula is as follows:

QFL(*σ*) = −|*y* – *σ*|*^β^* ((1 − *y*) log (1 − *σ*) +*y*log(*σ*)) (5)

GEL (*p_y_*_1_, *p_yr_*) = −|*y* − (*y*_1_*p_y_*_1_ + *y_r_p_yr_*)|*^β^* ((*y_r_* − *y*) log(*p_y_*_1_) + (*y* − *y_l_*) log(*p_yr_*)) (6)

*y* is the overall label of the detection target, and *y_r_* and *y_l_* are the true values of classification and regression. *p_y_*_1_ and *p_yr_* are the predicted values corresponding to the former, and *β* is the hyperparameter. Quality focal loss and distribution focal loss make up this function. Among them, quality focal loss uses hyperparameters to ensure the balance between the number of categories. Its formula is as follows: *σ* stands for the predicted value, and *y* stands for the quality label between 0 and 1.

## 3. Experiments

### 3.1. Experimental Platform

The experiments in this paper are completed in the environment of Table 1.

### 3.2. Dataset Description

**Figure 7:**
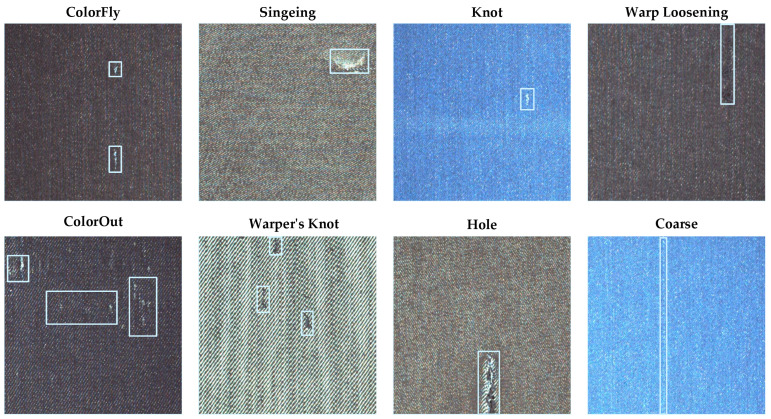
Examples of different types of fabric defects.

**Figure 8:**
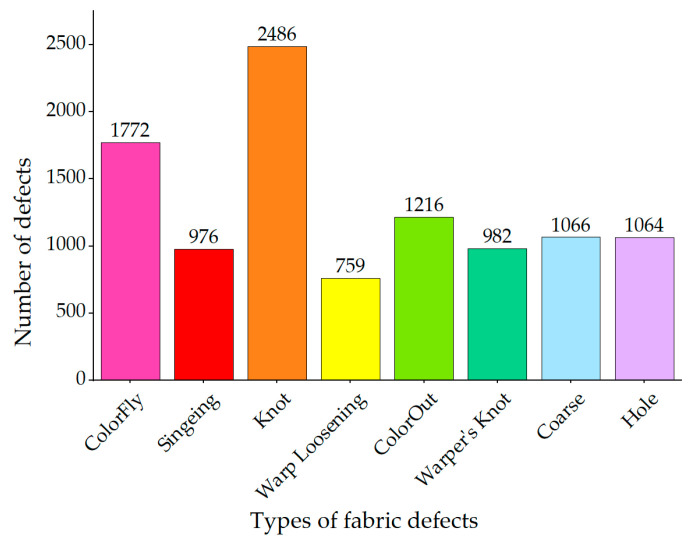
Statistics of fabric defect types.

The data set used in the experiment were taken by Alibaba Cloud Tianchi [30] in a textile workshop in Guangdong Province. After manual sorting and selection, 10,321 pictures were selected, including 8 kinds of defects. They are ColorFly, Singeing, Knot, Warp Loosening, ColorOut, Warper’s Knot, Hole, and Coarse. An example of each type of fabric defect is shown Figure 7. The specific number of fabric faults is shown in Figure 8.

**Figure 9:**
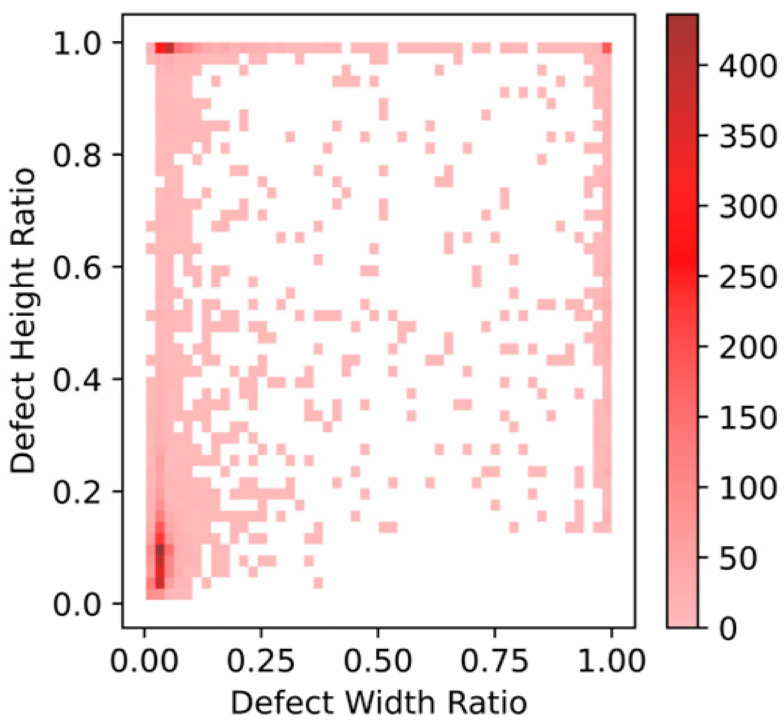
Proportion of defect size.

Figure 9 illustrates the proportion of fabric defects in the entire fabric area. It can be seen that in the fabric data set, most of the defects are small-sized defects, and the features are difficult to capture, and the aspect ratio of the defects is widely distributed, so detection is difficult.

For the sake of the training accuracy of the model and to prevent overfitting, this paper divides the fabric data set into training, validation, and test set. Its division proportion is 80%, 10%, and 10%.

**Figure 10:**
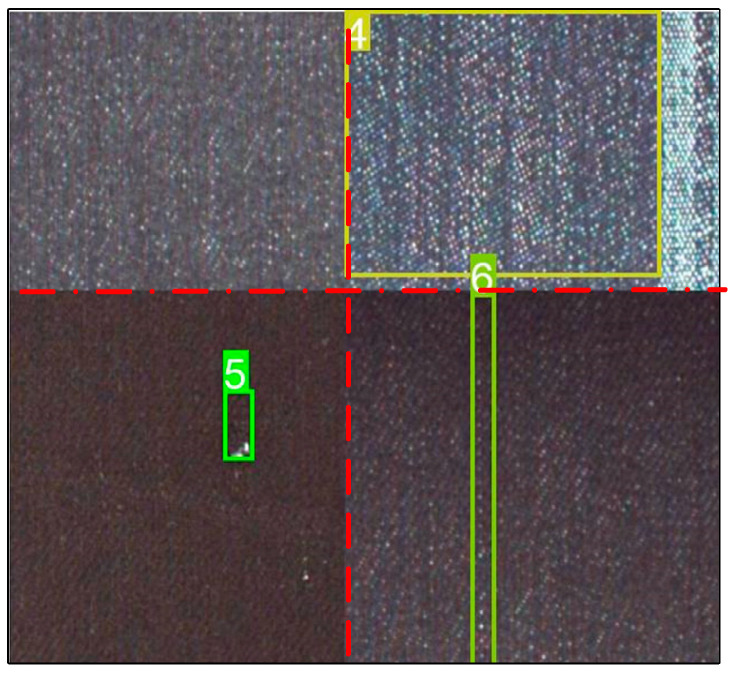
Mosaic data enhancement.

Due to the low number of certain kinds of defects in the original fabric dataset, it may cause underfitting of this type of defect. In this paper, methods such as flipping, zooming, adding noise, splicing, and mosaic enhancement are used to expand the number of some fabric images. The effect of mosaic data enhancement is shown in Figure 10. Combining four different fabric pictures enables the model to learn various types of features during each training, thereby improving the detection and generalization capabilities of the model, and consecutive effects of the imbalance in the number of fabrics.

### 3.3. Evaluation Indicators and Experiment

To demonstrate the effectiveness of the modified algorithm for fabric detection, this experiment adopts precision rate (P), recall rate (R), and mean average precision (mAP) as evaluation indicators of the model.

The mAP is an index to comprehensively measure the accuracy of model detection, and it is the most important index in target detection. Its specific calculation formula is as follows:

Precision = TP/(TP + FP)(7)

Recall = TP/(TP + FN)(8)

mAP = ∑AP/C(9)

TP is the number of correctly detected targets, FP is the number of falsely detected targets, FN is the number of missed detections, C is the total number of defect categories, and ∑AP is the sum of the precision values of all defect categories.

In addition, this experiment also introduces the frames per second (FPS) and the parameter size of the model as one of the evaluation indicators of the model performance. The higher the speed, the more it can satisfy the needs of real-time detection of fabrics.

In the training phase of this model, the input size of the fabric image is changed to 640 × 640, the initial learning rate is set to 0.0005, the optimizer selection is SGD, and the batch size is changed to 8.

**Figure 11:**
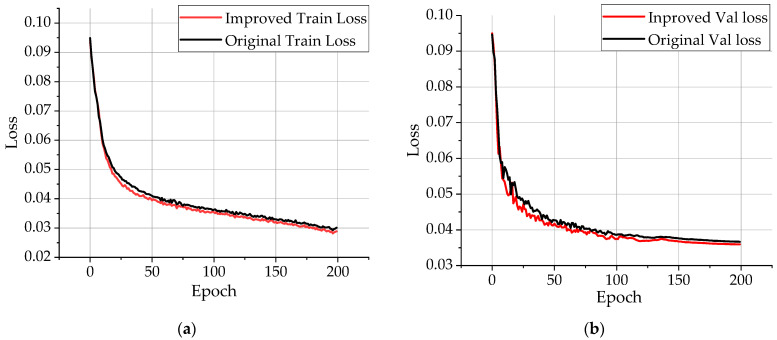
Loss function graph. (**a**) Train loss; (**b**) Val loss.

Figure 11 shows the variation of loss value with epoch during training of the benchmark network and the improved network. Based on the loss function graph, we know that the loss decreases rapidly during the 25 epochs of training, and the loss decreases gradually and becomes smooth after 150 epochs after training. It can be shown that the network training has not occurred overfitting. The loss of the improved model training and validation has a higher drop rate, indicating that the training effect of the improved model is better than the baseline model.

### 3.4. Loss Function Effect Verification

In order to solve the problem of fabric imbalance, this paper first performs mosaic data enhancement, translation, flip, and other operations on the data to improve the generalization of the network by increasing the number. Secondly, the generalized focal loss function is used to enhance the model’s learning of positive samples. In this section, focal loss and generalized focal loss are introduced in the benchmark network to explore the impact of the improvement of the loss function on the detection accuracy.

It can be seen from Table 2 that the generalized focal loss function introduced in this paper can improve the classification effect. The generalized focal loss function increases the learning weight of positive samples and reduces the learning weight of useless negative samples, so as to resist the imbalance caused by positive and negative samples. Secondly, the generalized focal loss function predicts the results through discretization to improve the value of the IoU.

### 3.5. Ablation Experiment

Through the ablation experiments on the improved algorithm structure, the effectiveness of our proposed modules and improvements in the performance of fabric defect detection networks is verified. In this paper, YOLOv5 is used as the benchmark network and the structure using Swin Transformer as the backbone and introducing the BiFPN and the small-target detection layer structure called YOLO-SB. On this basis, the network obtained by removing the BiFPN and introducing generalized focal loss is called YOLO-SL. On the basis of the BiFPN and small-target layer, the network that only introduces generalized focal loss is called YOLO-LB. The network introduced by all the improvements is called YOLO-TLB. The experimental results are shown in Table 3. The mAP results of different defect types are shown in Table 4.

From the comparison between the baseline network, we can see that after using the light Swin Transformer backbone network to replace the original backbone layer, the ability to reshape features and extract local features can be strengthened, and the detection performance of small fabric defects can be improved. Compared with FPN, the weighted bidirectional feature network can enhance the feature fusion between different scales, improve the reuse of features, and facilitate the detection of small fabric defects. On the second basis, by adding a detection layer for comparison, both the map and recall rates are increased, indicating that the new detection layer improves the detection effect of small pixel defects. Comparing the results by YOLOV5 and YOLO-TBL, the recall rate is greatly improved after adding the generalized focal loss function, and the map also increases. The network is fully trained on the positive samples, which alleviates the extremely imbalanced number of background samples and foreground samples. Finally, the improved method proposed has an mAP of 75.9% and a recall rate of 73.1% in fabric defect detection. The experiments show that the improved algorithm can achieve high-precision and high-efficiency fabric defect detection.

**Figure 12:**
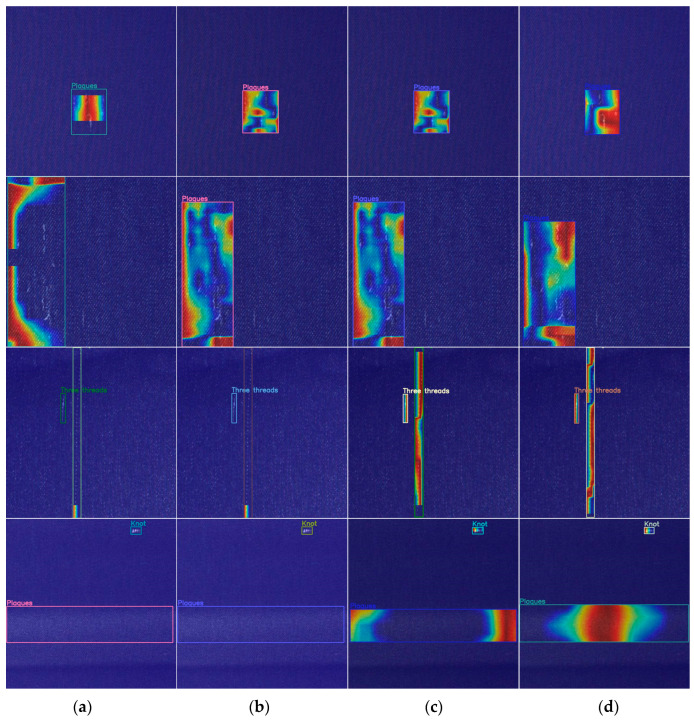
Heatmaps of different networks in ablation experiments: (**a**) CAM heat map of YOLO-LB; (**b**) CAM heat map of YOLO-SL; (**c**) CAM heat map of YOLO-SB; (**d**) CAM heat map of YOLO-TLB.

In addition, we use a gradient-free algorithm Eigen CAM [31] to generate a network activation heat map to visually show the effect of improving the ablation. The comparison of the CAM heat map of the ablation experiment results in this section is shown in Figure 12.

### 3.6. Ablation Experiments with Different Attention Mechanisms

**Figure 13:**
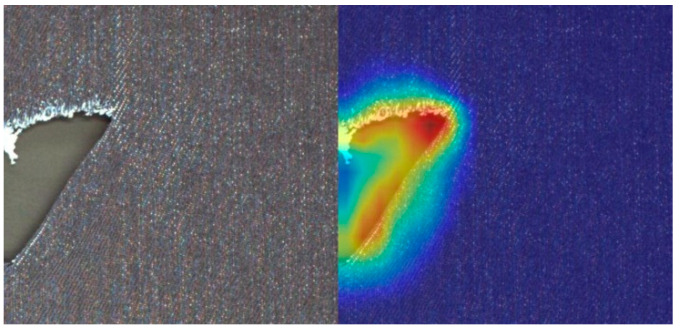
Attention of Eigen CAM heatmap.

To verify the improvement effect of the moving window-based mechanism proposed in this paper on the fusion of the YOLOv5 network model and other attention mechanisms, this paper adds several classic attention mechanisms to the neck layer for comparative experiments. Table 5 shows the experimental results of different attention. Figure 13 shows the effect of the CAM heatmap after adding the attention mechanism.

This attention mechanism can explicitly show the area that the model pays attention to focus on. From the experimental results, it can be seen that after adding the Transformer’s self-focus mechanism, mAP has increased, which can effectively strengthen the feature extraction capability of the model for low-resolution images, help to retain the feature information of fabric defects, and strengthen the network’s ability to detect features around small defects. After introducing the GAM attention to the neck, the extraction ability of small objects is further improved.

### 3.7. Results Visualization

**Figure 14:**
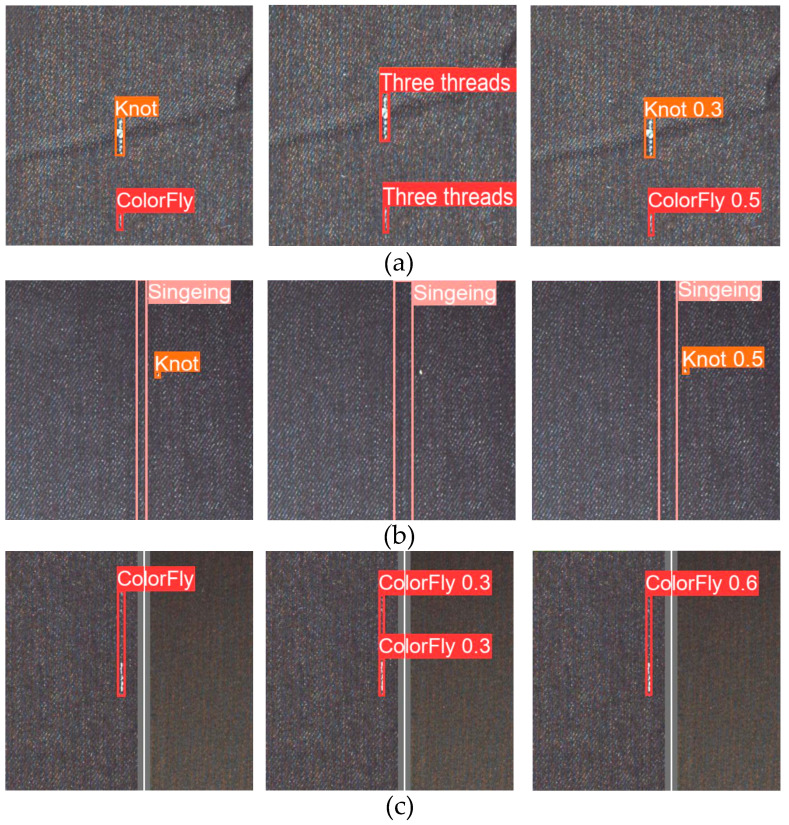
Some visual detection results. The first picture on the left is the labeled picture, the middle picture is the detection result of the original YOLOv5 algorithm, and the picture on the right is the detection result of the improved algorithm: (**a**) Detection result of the similar appearance of two kinds of fabric defects; (**b**) detection result of small target size defects; (**c**) detection result of overlapping fabric defects.

To more intuitively feel the detection effect of the modified method on defects, we choose several fabric images to compare the detection results. The left picture is the marked image, the middle picture is the unimproved YOLO algorithm detection picture, and the right picture is the modified algorithm detection picture. From Figure 14, the original YOLO algorithm to detect fabric defects has problems such as false detection of similar objects, missed detection of small defect objects, or poor detection effect of overlapping defects. The improved algorithm has a significantly improved detection effect.

### 3.8. Comparison with State-of-the-Art Methods

To further demonstrate the advantages of the modified model in this paper, different mainstream target detection networks are trained under the same dataset, and the method proposed in our paper is compared to five classical target detection algorithms under the same experiment platform and dataset. The experimental results are shown in Table 6.

Through the comparison of the test results of various classic target networks on the fabric defect data set, the improved Swin Transformer algorithm proposed in this paper achieves an average accuracy of mAP of 76.5% and an accuracy of 85.6%. Compared with other detection methods, the algorithm has certain advantages, and the number of detections per second reaches 58.8, which meets the needs of enterprises and the actual situation.

## 4. Conclusions

This paper takes fabric defects as the research object, and realizes the precise location and classification of fabric defects by improving the algorithm. The YOLOv5 model is used as the baseline network, and the Swin Transformer encoder is added to the backbone network of the fabric defect detection model. The multiwindow sliding self-attention is added to strengthen the weighted bidirectional feature network, and a detection layer that can detect 4 × 4 small targets is added to the four-scale detection to enhance the detection of small defects around the extraction of feature information. The generalized focal loss function is introduced to strengthen the algorithmic learning of positive sample instances and reduce the missed detection rate. After experimental comparison with the above five target detection algorithms, compared with the original frame, the modified fabric defect detection algorithm of the Swin Transformer proposed achieves 76.5% in the mAP and 58.8 FPS in the real-time detection speed, which meets the needs of enterprises.

In subsequent research, we will continue to optimize and improve the fabric defect method. Under the condition of keeping the detection accuracy basically unchanged, the number of model calculation parameters and time consumption or the fabric samples required for training should be reduced so that the model can achieve the same detection effect in embedded or mobile terminals.

## Figures and Tables

**Table 1:** The experimental environment.

Project	Hardware Specifications (Software Version)
operating system	Ubuntu18.04
CPU	AMD Ryzen5 5600X
GPU	NVIDIA GeForce RTX 3060TI
Software environment	Pytorch 1.7.0, Python3.9, OpenCV 4.6, CUDA 11.6, CuDNN 8.4.0

**Table 2:** Loss function optimization result.

Algorithm	mAP (%)
YOLO	72.3
YOLO + Focal Loss	72.4 (+0.1)
YOLO+ Generalized Focal Loss	72.8 (+0.5)

**Table 3:** Ablation experiment.

Algorithm	With Swin T	With Loss	With BiFPN	mAP@0.5	Recall	Weight (MB)
YOLO-LB		√	√	73.8	71.4	14.9
YOLO-SL	√	√		74.6	70.6	18.3
YOLO-SB	√		√	74.8	71.8	20.0
YOLO-TLB	√	√	√	75.9	73.1	20.1

**Table 4:** Results for each fabric defect category.

Defect Type	mAP@0.5				
	YOLOv5	YOLO-LB	YOLO-SL	YOLO-SB	YOLO-TBL
ColorFly	77.6	79.8	76.9	79.1	81.9
Singeing	60.3	68.8	67.5	64.9	66.7
Knot	72.7	68.2	65.8	73.1	73.7
Warp Loosening	58.5	61.7	66.8	66.8	62.6
ColorOut	88.0	89.1	91.2	89.6	91.3
Warper’s Knot	53.6	53.2	59.0	55.4	55.2
Hole	73.7	79.5	78.2	76.9	82.1
Coarse	92.7	90.2	91.3	92.8	93.7
All classes	72.2	73.8	74.6	74.8	75.9

**Table 5:** Results of different attention mechanisms.

Attention Mechanism Model	mAP (%)
YOLO-TLB	75.9
+CBAM [32]	76.1 (+0.2)
+SE [33]	75.1 (−0.8)
+GAM [34]	76.5 (+0.6)

**Table 6:** Comparison with state-of-the-art methods.

Algorithm	Backbone Network	Precision (%)	mAP (%)	FPS
SDD [35]	VGG16	50.6	40.2	83.3
Faster R-CNN [36]	ResNet50	76.2	65.9	12.5
YOLOv3-Tiny [37]	Darknet53	46.1	46.7	113.6
YOLOv4-Mish [38]	CSPDarknet	67.8	58.8	111.1
YOLOv5 [39]	CSPDarknet	77.0	72.1	90.9
OUR	Swin Transformer	85.6	76.5	58.8

## Data Availability

Not applicable.
